# Machine learning-based analysis and prediction of factors influencing mental health among children and adolescents in Jiangsu Province

**DOI:** 10.1186/s13034-025-00959-5

**Published:** 2025-08-31

**Authors:** Yiliang Xin, Yan Wang, Xiyan Zhang, Peixuan Li, Wenyi Yang, Bosheng Wang, Jie Yang

**Affiliations:** 1 Department of Child and Adolescent Health Promotion, Jiangsu Provincial Center for Disease Control and Prevention, Nanjing City, Jiangsu Province China; 2 Department of Occupational Disease Prevention and Control, Jiangsu Provincial Center for Disease Control and Prevention, Nanjing City, Jiangsu Province China

**Keywords:** Machine learning, Children and adolescents, Mental health, Predictive modeling

## Abstract

**Background:**

This study investigates the current mental health status among children and adolescents in Jiangsu Province by analyzing symptoms of depression, anxiety, and stress using standardized psychological scales. Machine learning models were utilized to identify key influencing variables and predict mental health outcomes, aiming to establish a rapid psychological well-being assessment framework for this population.

**Objective:**

A cross-sectional survey was conducted via random cluster sampling across 98 counties (cities/districts) in Jiangsu Province, enrolling 141,725 students (47,502 primary, 47,274 junior high, 11,619 vocational high school students, and 35,330 senior high ). The study focused on prevalent mental health disorders and associated risk factors.

**Methods:**

Depression, anxiety, and stress scores served as dependent variables, with 57 socio-demographic and behavioral factors as independent variables. Five supervised machine learning models (Decision Tree, Naive Bayes, Random Forest, K-Nearest Neighbors (KNN), and XGBoost) were implemented using R software. Model performance was evaluated using accuracy, precision, recall, F1 Score and Area Under the ROC Curve (AUC). Feature importance analysis was conducted to identify key predictors.

**Results:**

The study revealed significant mental health disparities: depression (14.9%), anxiety (25.5%), and stress (10.9%) prevalences showed clear gender and regional gradients. Females exhibited higher rates across all conditions (*p* < 0.05), and urban areas had elevated risks compared to suburban regions. Mental health deterioration escalated with educational stages (e.g., depression from 9.2% in primary to 21.2% in senior high; χ²_trend_ = 2274.55, *p* < 0.05). The XGBoost model demonstrated optimal predictive performance (AUC: depression = 0.799, anxiety = 0.770, stress = 0.762), outperforming other models. Feature importance analysis consistently identified bullying duration, age, and drinking history as top risk factors across both Gain and SHAP methods, while SHAP values additionally emphasized modifiable lifestyle factors (e.g., breakfast frequency) and demographic variables (e.g., gender).

**Conclusions:**

This study identifies bullying, age, and alcohol consumption history as key mental health risk factors among Jiangsu’s children and adolescents. These findings emphasize the need for school-based anti-bullying programs, age-specific mental health counseling, and healthy lifestyle education (including alcohol refusal). Lifestyle behaviors like daily breakfast intake should be integrated into dietary interventions for mental health promotion. Urban-rural and gender disparities necessitate targeted support for urban adolescent females, while educational stage differences highlight the criticality of early prevention.

## Introduction

The World Health Organization (WHO) defines mental health as a state of well-being encompassing emotional, psychological, and social dimensions, extending beyond the absence of disorders. China’s Statistical Report on Youth Development (2020) indicates that approximately 30 million individuals under 17 experience emotional or behavioral challenges. Globally, 25% of U.S. adolescents aged 12 ~ 17 used mental health services between 2013 and 2019 [1]. Adolescence represents a critical developmental phase where unresolved mental health issues may lead to long-term consequences for individuals, families, and society.

Depression is a prevalent mental disorder in youth, characterized by prolonged courses and high recurrence rates. Symptoms include persistent sadness, anhedonia, hopelessness, anxiety, and even suicidal ideation [2]. Onset often begins in childhood and escalates during adolescence [3]. Global studies suggest that approximately 20% of children and adolescents experience depression or related symptoms, with prevalence increasing over time [4]. Depression imposes enduring psychological and physical burdens [5], disrupting family dynamics and societal development [6], thus necessitating early identification and intervention as global health priorities [7].Stress refers to an individual’s subjective response to life challenges. Chronic stress is associated with dysregulated cortisol secretion, adversely affecting physical and mental health [8]. For youth, stressors primarily derive from academic pressures [9], parental expectations, peer interactions, and adolescent growth crises [10]. Prolonged stress exposure impairs neurological, physiological, and behavioral functioning [11, 12, 13] and serves as a precursor to anxiety [14]. Anxiety, the brain’s response to perceived threats [15], is the most prevalent mental disorder among adolescents, typically emerging during puberty [16]. It detrimentally impacts learning, life satisfaction, and physical health [17] and strongly correlates with comorbidities conditions like depression [18] and self-harm. Rising rates of anxiety, depression, and suicide among adolescents have been documented globally [19], with parental anxiety further exacerbating risks for offspring [20].

Study data were obtained from the Jiangsu Provincial Student Common Diseases and Health Influencing Factors Surveillance Project (Ethics Approval: 2023ZDSYLL456-P01). Guided by the biopsychosocial model as the core framework, we categorized variables into three major dimensions: individual biological characteristics (such as age, gender, weight, and waist circumference), psychobehavioral factors (including smoking and drinking history), and social environmental factors (e.g., school bullying and family structure). Concurrently, drawing on the social-ecological model, we incorporated nested influencing factors ranging from the micro-level (e.g., family structure) to the macro-level (e.g., urban-rural disparities), ensuring comprehensive coverage of the four interconnected levels of individual, family, school, and community. The selection of relevant variables is supported by existing literature. For demographic variables (age, gender, urban-rural differences, etc.): Patalay Praveetha et al. [21] through the Millennium Cohort Study, examined the mental health status of 9,553 children at two critical age stages (11 and 14 years old) and explored the role of gender differences in age-related changes in mental health. A study on the mental health of rural-to-urban migrant children in Guangzhou identified urban-rural disparities as a significant factor affecting the mental well-being of children and adolescents [22]. Regarding lifestyle variables (smoking, alcohol consumption, physical activity, dietary patterns, etc.): Wen-Yi Yang, et al. [23] found that smoking, drinking, low physical activity, and unhealthy diets significantly elevate the risk of depressive symptoms among middle school students. For school environmental variables (duration of bullying, being mocked, online rumors, etc.): Based on a longitudinal cohort study tracking adolescents aged 13–16 in northern Sweden, Evelina Landstedt, et al. [24] demonstrated associations between specific school environmental variables—such as bullying duration, mockery, and online rumors—and mental health issues like depression and anxiety in children and adolescents. In terms of family and health variables (family structure, myopia, etc.): Xin-Xin Huang et al. [25] analyzed the impact of different family structures (single-parent, three-generation, boarding, reconstituted families, etc.) on depressive and anxiety symptoms among students from 7 middle schools in Shanghai. A long-term follow-up study on Israeli adolescents (2011–2022) confirmed a dose-response relationship between the severity of myopia and mental health problems such as anxiety [26].

This study utilizes the Center for Epidemiological Studies Depression Scale (CES-D), Generalized Anxiety Disorder 7-item Scale (GAD-7), and Depression Anxiety Stress Scales-21 (DASS-21) to quantify mental health symptoms. Five machine learning models were applied to identify predictors of depression, anxiety, and stress.

## Materials and methods

### Data collection

The sample included 141,725 students from primary (grades 4–6), junior high, senior high and vocational schools across 98 counties. Sampling procedures involved random selection of seven schools in urban areas (2 primary, 2 junior high, 1 vocational, 2 senior high) and five schools per county (2 primary, 2 junior high, 1 senior high). Whole-class sampling was used, with ≥ 80 students per grade. Anomalous data were excluded prior to analysis.

### Mental health assessment tools

① Depression: CES-D Scale (20 items, 4-point Likert scale; total score 0 ~ 60). Scores ≥ 20 indicate depression.
② Anxiety: GAD-7 Scale (7 items, 4-point Likert scale; total score 0 ~ 21). Scores ≥ 5 indicate anxiety.
③ Stress: DASS-21 Stress Subscale (7 items, scores multiplied by 2; total score 0 ~ 42). Scores ≥ 15 indicate stress.

### Machine learning models

Five supervised models (Decision Tree, Naive Bayes, Random Forest, KNN, XGBoost) were trained in R.

① Decision Tree: method = “class”, split = “gini”, minsplit = 20, minbucket = 10, cp. = 0.001, maxdepth = 5.
② Naive Bayes: laplace = 1.
③ Random Forest: ntree = 100, mtry = floor(sqrt(ncol(train) − 1)), importance = TRUE.
④ KNN: tuneGrid = expand.grid(k = 5), metric = “Accuracy”, preProc = c(“center”, “scale”).
⑤ XGBoost: eta = 0.3, max_depth = 6, nrounds = 100, early_stopping_rounds = 10, verbose = 1.

Grid search with cross-validation was used to optimize hyperparameters. Model performance was evaluated using AUC, a key metric for binary classification tasks.

### Data processing

Inclusion criteria for independent variables in this study: Variables that demonstrated statistically significant associations (*p* < 0.05) with mental health outcomes (defined as depression = 1, anxiety = 1, or stress = 1). Categorical variables with missing values (e.g., drinking history, fresh fruits frequency, vegetables frequency, drinking milk frequency, breakfast frequency, days of exercise, physical education classes, being seriously injured, bullying-related variables, family type and parental myopia) were imputed using the “mice” package (method = “polyreg”) with three iterations of multiple imputation.

Data were split into training (70%) and testing (30%) sets via the “caret” package. Models were trained on the training set and evaluated on the testing set via confusion matrices, calculating accuracy, precision, recall, F1 score, and AUC.

### Feature importance analysis

Using the XGBoost model, the “importance( )” function in R extracted Gain values for 57 independent variables (e.g., age, gender, height, breakfast frequency, bullying duration). SHAP value analysis was conducted to validate result stability, identifying top features for each mental health outcome.

### Quality control

Prior to the initiation of this monitoring project, survey personnel underwent standardized training in monitoring methodologies and techniques. The monitoring activities were executed in strict accordance with the procedures and methods delineated in the work manual. Quality control personnel conducted on-site evaluations, and the data uploaded were subjected to logical review through the monitoring system.

### Statistical analysis

Data organization was conducted using Excel software, while data analysis was performed utilizing IBM SPSS Statistics 23.0 standard network version and R 4.4.2 software. Continuous variables are presented as mean ± standard deviation (SD), and categorical variables are expressed as frequencies (%). Differences between groups were evaluated using χ^2^ tests with a significance level of α = 0.05.

## Results

### Participant characteristics

The cohort (*N* = 141,725) had a mean age of 13.6 ± 2.6 years, with 52.1% males (73,888) and 55.8% urban residents. Educational distributions were 33.5% primary, 33.4% junior high, 24.9% senior high, and 8.2% vocational school students.

### Mental health prevalence

Depression: Overall prevalence was 14.9%, with females (16.0%) significantly higher than males (13.9%, χ^2^ = 131.61, *p* < 0.05). Urban areas had higher rates (15.7%) than suburban regions (13.9%, χ^2^ = 90.40, *p* < 0.05). Prevalence increased with education: 9.2% (primary) to 21.2% (senior high, χ^2^_trend_ = 2274.55, *p* < 0.05; Table 1).

**Table 1:** Depression detection of subjects [n(%)]

Variable	Positive	Negative	χ^2^	*p*-value
*Gender*			131.61	< 0.05
Male	10,236 (13.9)	63,652 (86.1)		
Female	10,871 (16.0)	56,966 (84.0)		
*Regions*			90.40	< 0.05
Urban	12,405 (15.7)	66,640 (84.3)		
Suburban	8,702 (13.9)	53,978 (86.1)		
*Educational stages*			2274.55^*^	< 0.05
Primary	4,363 (9.2)	43,139 (90.8)		
Junior high	7,100 (15.0)	4,0174 (85.0)		
Vocational	2,142 (18.4)	9,477 (81.6)		
Senior high	7,502 (21.2)	2,7828 (78.8)		
Total	21,107(14.9)	120,618(85.1)		

*Trend chi-square value

Anxiety: Overall prevalence was 25.5%, with females (29.1%) higher than males (22.1%, χ²=910.64, *p* < 0.05). Urban areas had higher rates (26.6%) than suburban regions (24.1%, χ²=112.55, *p* < 0.05). Prevalence escalated from 15.2% (primary) to 38.8% (senior high, χ^2^_trend_ = 5387.21, *p* < 0.05; Table 2).

**Table 2:** Anxiety detection of subjects [n(%)]

Variable	Positive		Negative	χ^2^	*p*-value
*Gender*				910.64	< 0.05
Male	16,352 (22.1)		57,536 (77.9)		
Female	19,756 (29.1)		48,081 (70.9)		
*Regions*				112.55	< 0.05
Urban	21,003 (26.6)		58,042 (73.4)		
Suburban	15,105 (24.1)		47,575 (75.9)		
*Educational stages*				5387.21^*^	< 0.05
Primary	7,212 (15.2)		40,290 (84.8)		
Junior high	1,2078 (25.5)		35,196(74.5)		
Vocational	3,103 (26.7)		8,516 (73.3)		
Senior high	13,715 (38.8)		21,615 (61.2)		
Total	36,108 (25.5)		105,617 (74.5)		

*Trend chi-square value

Stress: Overall prevalence was 10.9%, with females (11.9%) higher than males (10.0%, χ²=128.67, *p* < 0.05). Urban areas had marginally higher rates (11.2%) than suburban regions (10.5%, χ²=21.77, *p* < 0.05). Prevalence increased from 8.2% (primary) to 14.5% (senior high, χ²=870.15, *p* < 0.05; Table 3).

**Table 3:** Stress detection of subjects [n(%)]

Variable	Positive	Negative	χ^2^	*p*-value
*Gender*			128.67	< 0.05
Male	7391(10.0)	66,497(90.0)		
Female	8061(11.9)	59,776(88.1)		
*Regions*			21.77	< 0.05
Urban	8890(11.2)	70,155(88.8)		
Suburban	6562(10.5)	56,118(89.5)		
*Educational stages*			870.15	< 0.05
Primary	3908(8.2)	43,594(91.8)		
Junior high	5375(11.4)	41,899(88.6)		
Vocational	1050(9.0)	10,569(91.0)		
Senior high	5199(14.5)	30,211(85.5)		
Total	15,452(10.9)	126,273(89.1)		

### Machine learning model performance

Utilizing the determination results of depression, anxiety, and stress as dependent variables, alongside 57 study variables as independent variables, we applied five machine learning models implemented in R to predict the positive outcomes of depression, anxiety, and stress among the study participants. The performance of these models was evaluated through the construction of confusion matrices, as presented in Tables 4, 5 and 6.

**Table 4:** Evaluation metrics of five models for depression

Evaluation metrics	Decision Tree	Naive Bayes	Random Forest	KNN	XGBoost
Accuracy	0.862	0.813	0.862	0.851	0.864
Precision	0.869	0.905	0.871	0.870	0.876
Recall	0.983	0.871	0.985	0.971	0.979
F1 score	0.923	0.888	0.924	0.918	0.925
AUC	0.626	0.776	0.787	0.675	0.799

**Table 5:** Evaluation metrics of five models for anxiety

Evaluation metrics	Decision Tree	Naive Bayes	Random Forest	KNN	XGBoost
Accuracy	0.773	0.718	0.777	0.748	0.779
Precision	0.793	0.835	0.794	0.784	0.798
Recall	0.942	0.775	0.947	0.913	0.943
F1 score	0.861	0.804	0.864	0.844	0.865
AUC	0.699	0.738	0.762	0.670	0.770

**Table 6:** Evaluation metrics of five models for stress

Evaluation metrics	Decision Tree	Naive Bayes	Random Forest	KNN	XGBoost
Accuracy	0.893	0.850	0.894	0.886	0.894
Precision	0.896	0.924	0.896	0.898	0.900
Recall	0.996	0.906	0.996	0.984	0.990
F1 score	0.943	0.915	0.944	0.939	0.943
AUC	0.626	0.746	0.749	0.623	0.762

**Fig. 1:**
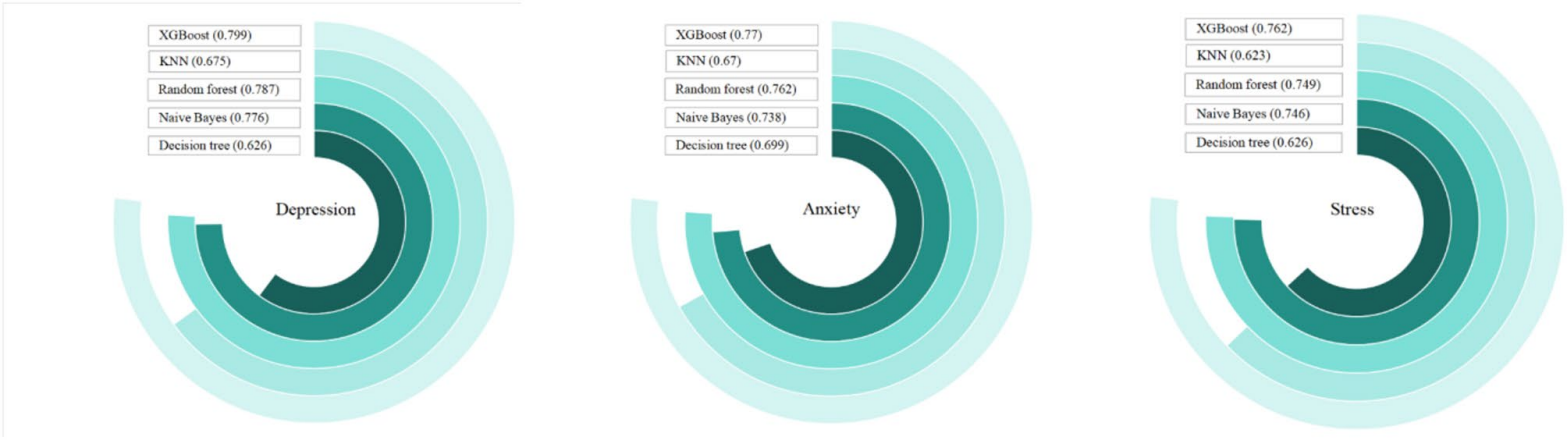
AUC value of five machine learning models

XGBoost outperformed other models for all outcomes: depression: AUC = 0.799, accuracy = 0.864, F1 = 0.925; anxiety: AUC = 0.770, accuracy = 0.779, F1 = 0.865; stress: AUC = 0.762, accuracy = 0.894, F1 = 0.943 (Tables 4, 5 and 6; Fig. 1). These results indicate that the XGBoost model is particularly well-suited for predicting positive cases of depression, anxiety, and stress in this study population.

### Feature importance

**Fig. 2:**
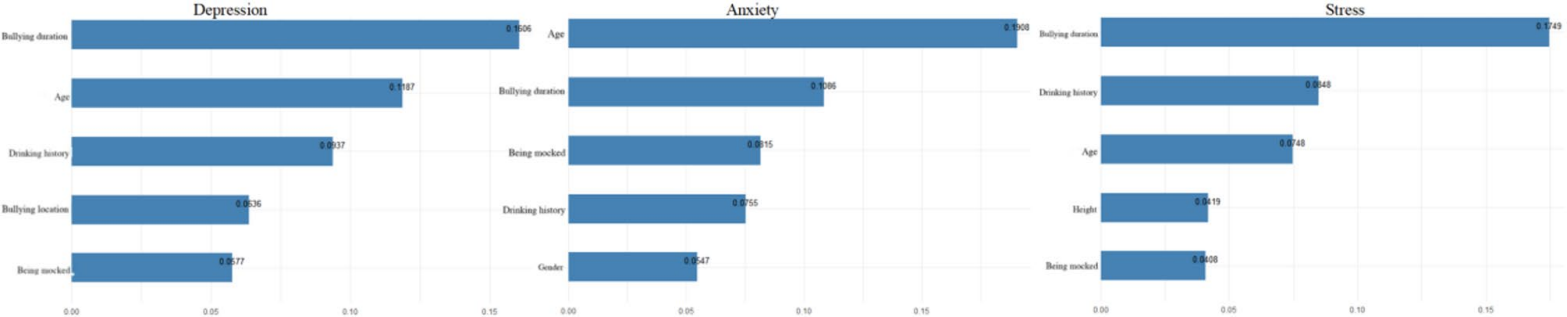
Top 5 feature by Gain Importance

The “importance” function of R software was applied to extract the Gain values affecting depression, anxiety and stress of subjects, and the top 5 feature variables were obtained, as shown in Fig. 2:

① Depression: bullying duration, age, drinking history, bullying location, being mocked;
② Anxiety: age, bullying duration, being mocked, drinking history, gender;
③ Stress: bullying duration, drinking history, age, height, being mocked.

**Fig. 3:**
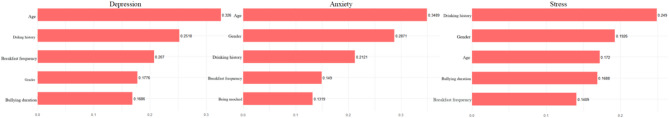
Top 5 feature by SHAP Importance

To validate the stability of the results, SHAP value-based feature importance analysis was conducted using R software, and the top five important feature variables for depression, anxiety, and stress were extracted, as shown in Fig. 3:

① Depression: age, drinking history, breakfast frequency, gender, bullying duration;
② Anxiety: age, gender, drinking history, breakfast frequency, being mocked;
③ Stress: drinking history, gender, age, bullying duration, breakfast frequency.

## Discussion

Current research on the mental health issues of student populations predominantly focuses on college students [27, 28], while relatively less attention is paid to the mental health of primary and secondary school students. Our study, leveraging data from the Jiangsu Provincial Student Common Diseases and Health Influencing Factors Surveillance Project, analyzed a representative sample of 141,725 primary and secondary school students, providing critical insights into adolescent mental health in Jiangsu Province. Our findings revealed that female adolescents exhibited significantly higher positive detection rates of depression, anxiety, and stress than males, aligning with previous studies by Keyes et al. and Shorey et al. [29, 30]. This gender disparity may reflect heightened psychological sensitivity in females, increasing their vulnerability to psychosocial stressors. Notably, urban students exhibited higher mental health risks than their suburban/rural counterparts, likely attributed to academic competition (e.g., college enrollment pressure), parental expectation pressure, and reduced outdoor activity [31]. Furthermore, the positive detection rate for mental health issues increases with the educational stages, emphasizing the necessity for targeted interventions, particularly semester-based assessments for urban female high school students.

A comparative analysis of five machine learning algorithms revealed distinct strengths and limitations: Decision Tree constructs hierarchical classification rules through recursive feature splitting, offering high interpretability and proficiency in handling nonlinear relationships. However, it is prone to overfitting and sensitive to class imbalance [32]. Naive Bayes applies Bayes’ theorem under the assumption of feature independence, excelling in computational efficiency and small-scale data scenarios. Its performance heavily relies on the validity of independence assumptions, which may degrade with irrelevant features [33]. Random Forest employs ensemble learning with bootstrap aggregating (bagging) and random feature subsets, enhancing robustness and reducing overfitting. Trade-offs include reduced interpretability and increased computational cost for large datasets [34]. K-Nearest Neighbors (KNN) classifies instances based on proximity to k neighbors, requiring no explicit training and adapting well to local patterns. Nevertheless, it exhibits high computational complexity and sensitivity to data scaling and outliers [35]. XGBoost utilizes gradient boosting with regularization, achieving state-of-the-art accuracy in capturing complex interactions. However, it demands rigorous hyperparameter tuning to prevent overfitting in high-dimensional settings [36].

In predicting depression, anxiety, and stress outcomes, performance metrics varied across algorithms. Depression: XGBoost achieved the highest AUC (0.799) and F1 score (0.925), balancing precision (0.885) and recall (0.965). Naive Bayes exhibited the highest precision (0.905), while Random Forest showed superior recall (0.985). Anxiety: XGBoost led in AUC (0.770) and F1 score (0.865), with competitive accuracy (0.779). Naive Bayes achieved the highest precision (0.835), and Random Forest excelled in recall (0.947). Stress: XGBoost outperformed in AUC (0.762) and accuracy (0.894), with F1 score (0.865) matching Decision Tree. Naive Bayes demonstrated the highest precision (0.924), while Decision Tree and Random Forest tied for recall (0.996).

Notably, while XGBoost emerged as the optimal model across primary metrics (AUC and accuracy), no single algorithm dominated all secondary metrics. This highlights the importance of metric-specific model selection and underscores the need for future research to explore: Advanced hyperparameter optimization techniques (e.g., Bayesian optimization), Hybrid modeling approaches integrating strengths of complementary algorithms.

Age, drinking history, and bullying duration emerged as consistent predictors across Gain and SHAP analyses. Older adolescents face compounding academic and developmental pressures [37], while alcohol use—shown to increase with age in longitudinal studies [38]—disrupts central nervous system function and exacerbates negative emotions. School bullying behaviors (“bullying duration” and “being mocked”) were strongly linked to psychological distress, corroborating global evidence [39, 40]. Notably, 16.7% of adolescents in Shantou, China, reported traditional bullying and 9.0% cyberbullying [41], with such experiences linked to subsequent violent behaviors and bidirectional relationships with pre-existing mental health conditions [42, 43]. However, enhanced self-esteem may mitigate bullying impacts [44].

Additionally, the Gain importance highlights that height is a significant factor affecting stress in children and adolescents (t-test: stress-positive individuals 160.25 ± 11.93 cm vs. 158.71 ± 12.49 cm, *p* < 0.05), possibly due to increased social attention, though conflicting evidence suggests short stature may also correlate with mental health issues [45]. Notably, SHAP analysis identified breakfast frequency as a key modifiable factor, aligning with studies linking irregular breakfast habits to mental health risks [46]. This suggests that interventions for the mental health of children and adolescents can be implemented by promoting improvements in healthy behaviors. Furthermore, Body metrics (weight, waistline) and online rumors further emerged as predictors, highlighting the impact of weight stigma and cyberbullying [47, 48].

For intervention, evidence supports physical activity [49], cognitive-behavioral therapy (CBT), and fluoxetine [50] for treating youth depression/anxiety. Our findings support multi-faceted interventions: anti-bullying programs, age-tailored counseling, dietary interventions to promote regular breakfast habits, and targeted support for urban female adolescents. Integrating machine learning-based screening with traditional assessments could enhance early detection.

Our study does have several limitations, this surveillance enrolled 141,725 primary and secondary school students in Jiangsu Province. Notwithstanding training for surveillance staff, variable proficiency in quality control methods may have introduced measurement variability. Additionally, the lengthy questionnaire (with numerous items) potentially induced inattentive responding—both factors compromising result accuracy. We selected 57 independent variables, all statistically associated with mental health. However, feature importance analysis revealed “bullying duration”, “age”, “gender”, “drinking history”, “bullying location”, and “being mocked” as top predictors, while other variables showed marginal significance. Including excessive variables posed multicollinearity risks, undermining predictive model validity. This underscores the need for future variable selection to integrate methods like LASSO regression—transcending univariate significance testing—to mitigate multicollinearity and prevent overfitting. And this study lacked direct measures of academic stress, parental expectations, and family socioeconomic status—variables for future questionnaires. Methodologically, exploring additional machine learning frameworks (e.g., neural networks, Transformer) and optimization strategies is warranted. The “black-box” nature of machine learning models hinders interpretability, necessitating caution in relying solely on predictions.

## Data Availability

No datasets were generated or analysed during the current study.
